# *Chlamydia trachomatis* GlgA Is Secreted into Host Cell Cytoplasm

**DOI:** 10.1371/journal.pone.0068764

**Published:** 2013-07-24

**Authors:** Chunxue Lu, Lei Lei, Bo Peng, Lingli Tang, Honglei Ding, Siqi Gong, Zhongyu Li, Yimou Wu, Guangming Zhong

**Affiliations:** 1 Department of Pathogen Biology, University of South China, Hengyang, Hunan, China; 2 Department of Microbiology and Immunology, University of Texas Health Science Center at San Antonio, San Antonio, Texas, United States of America; Institut Pasteur-URACNRS2582, France

## Abstract

Glycogen has been localized both inside and outside *Chlamydia trachomatis* organisms. We now report that *C. trachomatis* glycogen synthase (GlgA) was detected in both chlamydial organism-associated and -free forms. The organism-free GlgA molecules were localized both in the lumen of chlamydial inclusions and in the cytosol of host cells. The cytosolic GlgA displayed a distribution pattern similar to that of a known *C. trachomatis*-secreted protease, CPAF. The detection of GlgA was specific since the anti-GlgA antibody labeling was only removed by preabsorption with GlgA but not CPAF fusion proteins. GlgA was detectable at 12h and its localization into host cell cytosol only became apparent at 24h after infection. The cytosolic localization of GlgA was conserved among all *C. trachomatis* serovars. However, the significance of the GlgA secretion into host cell cytoplasm remains unclear since, while expression of chlamydial GlgA in HeLa cells increased glycogen stores, it did not affect a subsequent infection with *C. trachomatis*. Similar to several other *C. trachomatis*-secreted proteins, GlgA is immunogenic in women urogenitally infected with *C. trachomatis*, suggesting that GlgA is expressed and may be secreted into host cell cytosol during *C. trachomatis* infection in humans. These findings have provided important information for further understanding *C. trachomatis* pathogenic mechanisms.

## Introduction

Urogenital tract infection with *Chlamydia trachomatis* is a leading cause of sexually transmitted bacterial diseases [1–3]. However, the molecular mechanisms of *C. trachomatis* pathogenicity remain unclear. Nevertheless, the intracellular survival and replication of *C. trachomatis* organisms are thought to contribute significantly to inflammatory pathologies induced by *C. trachomatis* infection [4–7]. The *C. trachomatis* organisms have evolved a unique intracellular growth cycle with distinct biphasic stages [8]. The organisms invade epithelial cells via induced endocytosis in the form of elementary bodies (EBs). An endocytosed EB rapidly develops into a noninfectious but metabolically active reticulate body (RB) that is able to undergo biosynthesis and multiplication within the initially established cytoplasmic vacuole called chlamydial inclusion. The accumulation of progeny RBs in the inclusions triggers the differentiation of RBs back into EBs for exiting infected cells and spreading to new cells. The question is how chlamydial organisms are able to establish and maintain such a successful intracellular infection. The *C. trachomatis* organisms have evolved the ability to secrete proteins into both the inclusion membranes and host cell cytosol and the secreted proteins have been hypothesized to play important roles in modifying host cellular processes for facilitating chlamydial invasion, intracellular survival/replication and spreading to new cells [5,9–22]. Thus, searching for new *Chlamydia trachomatis*-secreted proteins (CtSPs) has been a most promising/productive approach for understanding chlamydial pathogenic mechanisms.

The *C. trachomatis* inclusions are known to contain glycogen that is detectable with iodine [23,24], which is consistent with the fact that the *C. trachomatis* genome encodes all necessary open reading frames (ORFs) required for both glycogen biosynthesis and catabolism [25]. Thus, the *C. trachomatis* organisms can both synthesize and utilize glycogen, which seems to be important in chlamydial pathogenesis since the plasmidless chlamydial organisms that are unable to produce a large amount of glycogen are no longer able to induce pathology in mouse oviduct [26]. Surprisingly, under electron microscopy, most glycogen particles were observed in the lumen of chlamydial inclusion [27,28], suggesting that most chlamydial glycogens are released out of the chlamydial organisms after/during synthesis or even synthesized outside of the organisms. It will be interesting to know whether the glycogen metabolism enzymes are also released into the inclusion lumen.

In the current study, we found that of the 6 glycogen metabolism-related enzymes investigated in the current study, only GlgA was detected outside of chlamydial organisms. An anti-GlgA antibody detected signals both inside and outside of chlamydial inclusions. Confocal microscopic analyses revealed that some intra-inclusion GlgA signals were completely independent of chlamydial organisms, suggesting that a portion of GlgA is secreted out of the organisms into the inclusion lumen. The extra-inclusion GlgA signal displayed a pattern similar to that of the *C. trachomatis*-secreted protease CPAF, suggesting that GlgA is also secreted into host cell cytosol. Efforts were made to ensure the antibody labeling specificities. GlgA secretion into host cell cytosol is highly conserved among all *C. trachomatis* serovars tested and may take place during infection in humans since GlgA is immunogenic in women urogenitally infected with *C. trachomatis* but not in rabbits immunized with dead *C. trachomatis* organisms, which is consistent with the concept that secretion of chlamydial proteins into host cell cytosol is often accompanied with enhanced immunogenicity. The above observations together have provided new information and tool for mapping the molecular basis of *C. trachomatis* pathogenesis.

## Materials and Methods

### 1: Chlamydial infection

The following *C. trachomatis* organisms were used in the current study: *C. trachomatis* serovars A/HAR-13 (ATCC catalog# VR-571B), B/HAR-36 (VR-573), Ba/Ap-2 (VR-347), C/UW-1, D/UW-3/Cx (VR-885), E/UW-5/CX, F/IC-Cal-3 (VR-346), G/UW-57/Cx (VR878), H/UW-43/Cx (VR-879), I/UW-12/Ur (VR-880), K/UW-31/Cx (VR-887), L1/LGV-440 (VR-901B), L2/LGV-434/Bu (VR-902B) & L3/LGV-404 (VR-903). These organisms were either purchased from ATCC (Manassas, VA) or acquired from Dr. Harlan Caldwell at the Rocky Mountain Laboratory, NIAID/NIH (Hamilton, MT) or Dr. Ted Kou at the University of Washington (Seattle, WA) or Dr. Li Shen at the Louisiana State University. The chlamydial organisms were propagated, purified, aliquoted and stored as described previously [17,22]. All chlamydial organisms were routinely checked for mycoplasma contamination. For infection, HeLa cells (human cervical carcinoma epithelial cells, ATCC cat# CCL2) grown in either 24 well plates with coverslips or tissue flasks containing DMEM (GIBCO BRL, Rockville, MD) with 10% fetal calf serum (FCS; GIBCO BRL) at 37°C in an incubator supplied with 5% CO_2_ were inoculated with chlamydial organisms. The infected cultures were processed at various time points after infection for either immunofluorescence assays, glycogen measurement or Western blot analysis as described below.

### 2: Chlamydial gene cloning, fusion protein expression and antibody production

The ORFs CT042 (GlgX), CT087 (MalQ), CT248 (GlgP), CT295 (MrsA), CT489 (GlgC), CT798 (GlgA), CT110 (HSP60), CT681 (MOMP), CT858 (CPAF; ref [22]:) CT795 (a secreted protein; ref [20]:), CT813 (an inclusion membrane protein; ref [29]:) and Pgp3 (a secreted plasmid protein; ref [18]:) from *C. trachomatis* serovar D genome (http://stdgen.northwestern.edu) were cloned into pGEX vectors (Amersham Pharmacia Biotech, Inc., Piscataway, NJ) and expressed as glutathione-s-transferase (GST) fusion proteins as described previously [30,31]. Expression of the fusion protein was induced with isopropyl-beta-D-thiogalactoside (IPTG; Invitrogen, Carlsbad, CA) and the fusion proteins were extracted by lysing bacteria via sonication in a Triton-X100 lysis buffer (1% Triton X-100, 1mM PMSF, 75 units/ml of Aprotinin, 20 µM Leupeptin and 1.6 µM Pepstatin, all from Sigma). After a high-speed centrifugation to remove debris, the fusion protein-containing supernatants were used for ELISA in glutathione-coated microplates or absorbed onto glutathione-conjugated agarose beads (Pharmacia) for antibody absorption experiment or further protein purification. The purified fusion proteins were used to immunize mice for producing antibodies as described previously [31].

### 3: Enzyme-linked immunosorbent assay (*ELISA*)

The fusion protein microplate ELISA was carried out as described previously [31]. Briefly, the GST-fusion proteins in the form of bacterial lysates were applied to glutathione-coated 96-well microplates (catalog number 15140B; Pierce, Rockford, IL) and used to assay antibody reactivities. All primary antibodies were preabsorbed with a bacterial lysate containing GST alone before they were assayed on the ELISA plates. The human and rabbit antisera were obtained and produced as described previously [30–32]. The goat anti-human IgA-IgG-IgM or donkey anti-rabbit IgG secondary antibodies conjugated with horseradish peroxidase (HRP) (catalog numbers 109-035-064 and 711-035-152, respectively; Jackson ImmunoResearch Laboratories, Inc., West Grove, PA) were used to probe the primary antibody binding. The soluble substrate 2,2′-azino-bis(3-ethylbenzothiazoline-6-sulfonic acid) diammonium salt (ABTS; catalog number A1888-5G; Sigma) was used to visualize the reactions, and the reactivity was recorded as the absorbance (optical density [OD]) at 405 nm. A GST-alone bacterial lysate-coated well in each plate was used as a negative control. Any wells with an OD equal to or greater than the mean plus 2 standard deviations were considered positive.

### 4: Immunofluorescence assay

The immunofluorescence assay was carried out as described previously [33,34]. HeLa cells with or without *C. trachomatis* infection grown on coverslips were processed for immunofluorescence assay. For some experiments, HeLa cells were transfected with the mammalian expression vector pFLAG-CMV4 (cat#E7158, Sigma) alone or recombinant pFLAG plasmid that encodes chlamydial GlgA gene. The GlgA gene from *C. trachomatis* serovar D was cloned into pFLAG-CMV4 using following primers: forward primer 5′-AAGGAAAAAA-GCGGCCGCG-(NotI)-ATGAAAATTATTCACACAGCTATC-3′ and back primer 5′-CC-GATATC-(ECoRV)-TTATTGTTTATAAATTTCTAAATATTTAT-3′. The transfection was carried out using the Lipofectamine 2000 following the manufacturer’s protocol (Invitrogen). Twenty-four hours after transfection, Flag-CT798 fusion protein was detected using an immunofluorescence assay or the transfected cultures were further infected with *Chlamydia trachomatis* organisms. For immunofluorescence assay, all cell samples were fixed with 2% paraformaldehyde (Sigma, St. Luis, MO) dissolved in PBS for 30 min at room temperature, followed by permeabilization with 2% saponin (Sigma) for an additional 30 min. After washing and blocking, the cell samples were subjected to antibody and chemical staining. Hoechst (blue, Sigma) was used to visualize DNA. A rabbit anti-chlamydial organism antibody (R1L2, raised with *C. trachomatis* L2 organisms, unpublished data) plus a goat anti-rabbit IgG secondary antibody conjugated with Cy2 (green; Jackson ImmunoResearch Laboratories, Inc) was used to visualize chlamydial organisms. The various mouse antibodies plus a goat anti-mouse IgG conjugated with Cy3 (red; Jackson ImmunoResearch) were used to visualize the corresponding antigens. The mouse antibodies included: polyclonal antibodies (pAbs) made against GST-GlgA, mAb 100a against CPAF [22], mAb clone 2H4 against Pgp3 [18] and mAb M1 against FLAG tag. All primary antibodies were pre-absorbed with a bacterial lysate containing GST alone before use. In addition, for some experiments, the primary antibodies were further absorbed with either the corresponding or heterologous fusion proteins immobilized onto glutathione-conjugated agarose beads prior to staining, which was meant to prove the antibody binding specificities. The absorption was carried out by incubating the antibodies with bead-immobilized antigens for 1h at room temperature (RT) or overnight at 4^o^C followed by pelleting the beads. The remaining supernatants were used for immunostaining. The immunofluorescence images were acquired using an Olympus AX-70 fluorescence microscope equipped with multiple filter sets and Simple PCI imaging software (Olympus, Melville, NY) as described previously [22]. For some experiments, the immunostaining was analyzed with confocal microscopy at UTHSCSA imaging core facility. The images were processed using Adobe Photoshop (Adobe Systems, San Jose, CA).

### 5: Western blot assay

The Western blot assay was carried out as described elsewhere [30]. Briefly, HeLa cells with or without *C. trachomatis* infection and with or without fractionation along with GST fusion proteins were resolved in SDS-polyacrylamide gels. The resolved protein bands were transferred to nitrocellulose membranes for antibody detection. The primary antibodies were: mouse polyclonal antiserum against GlgA (current study), mAb 100a against CPAF [22], mAb MC22 against chlamydial major outer membrane protein (MOMP; ref: [22]), mAb AF1 against the chlamydial inclusion membrane protein CT813 [29] and mAb W27 against host cell HSP70 (cat#Sc-24, Santa Cruz Biotechnology, CA), The anti-MOMP antibody was used to ensure that all lanes with chlamydial organism-containing samples had equivalent amounts of the organisms loaded while the lanes without chlamydial organism samples should be negative for MOMP. The anti-HSP70 antibody was used to make sure that an equal amount of normal HeLa and chlamydia-infected HeLa samples were loaded. The primary antibody binding was probed with an HRP (horse radish peroxidase)-conjugated goat anti-mouse IgG secondary antibody (Jackson ImmunoResearch) and visualized with an enhanced chemiluminescence (ECL) kit (Santa Cruz Biotech).

### 6: Glycogen quantification

Glycogen was quantified using the EnzyChrom^TM^ Glycogen Assay kit (cat#: E2GN-100, BioAssay systems, Hayward, CA) following a protocol recommended by the manufacturer. Briefly, HeLa cells grown in 6-well plates were infected with *C. trachomatis* L2 or transfected with pFLAG-CMV4 vector alone or recombinant pFLAG-CMV4-GlgA. 40h after infection or 24h after transfection, the cells were washed with ice-cold PBS and harvested in 0.5ml 10% KOH in 1.5ml microcentrifuge tube. After boiled at 100°C for 20 minutes and cooled to room temperature, Tricholoroacetic acid (TCA) was added to a final concentration of 10%. After centrifuging at 10,000g for 10min, the supernatant was transferred to a new tube and mix with 1ml ethanol and centrifuge at 4,000 g for 15min. The pellet was washed with 70% ethanol and air-dried. The precipitate was resuspended in 100 µl of distilled water. The concentration of glycogen in suspension was determined using the Glycogen Assay Kit. The final results were expressed as the total amount of glycogen per sample or well.

## Results

### 1: GlgA is detected in both chlamydial inclusion lumen and host cell cytosol

**Figure 1:**
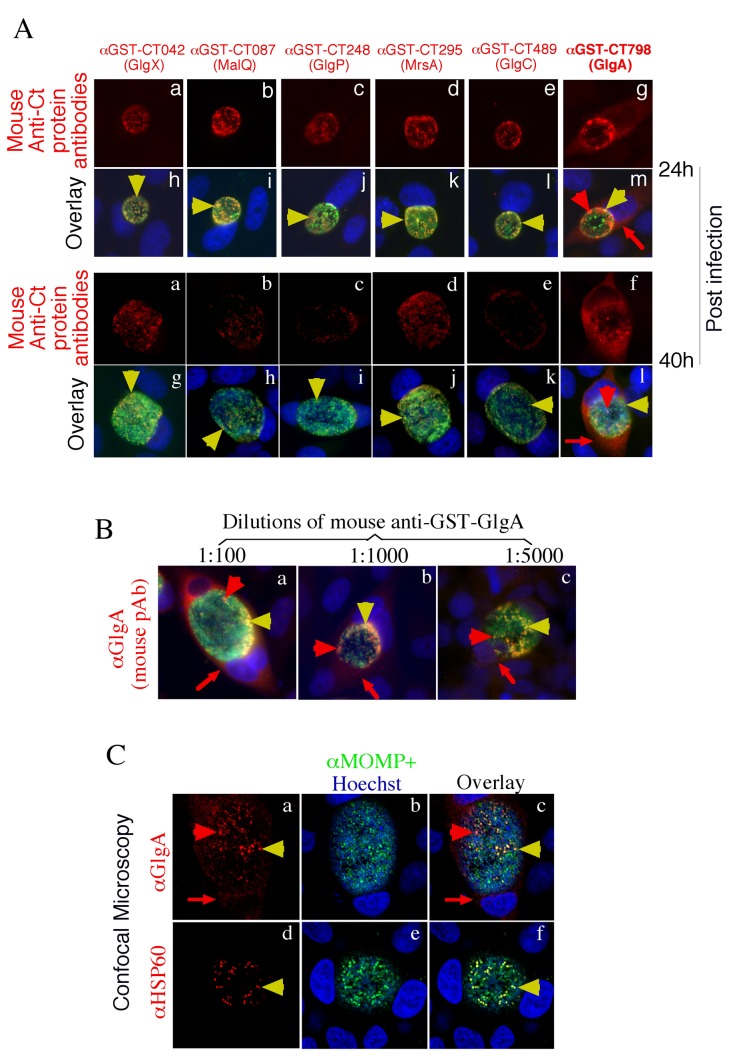
Localization of GlgA in *C. trachomatis*-infected cells. HeLa cells infected with *C. trachomatis* serovar L2 at an MOI 0.5 were processed either 24h or 40h post infection for co-staining with mouse antibodies recognizing individual chlamydial proteins visualized with a goat anti-mouse IgG conjugated with Cy3 (red), a rabbit antibody to chlamydial organisms visualized with a Cy2-conjugated goat anti-rabbit IgG (green) and DNA dye Hoechst (blue). (A) The mouse antisera raised with 6 GST fusion proteins as indicated on top of each panel were used to localize the corresponding endogenous chlamydial proteins after 1:1000 dilution. Note that only the anti-GST-CT798 (GlgA; panels f & l) detected signals that were free of chlamydial organisms. (B) The anti-GlgA antiserum labeling was repeated at multiple dilutions: 1:100 (panel a), 1:1000 (b) and 1:5000 (c). Note that the antibody detected both intra-inclusion (yellow & red arrowheads) and extra-inclusion (red arrow) signals under a conventional fluorescence microscope at all dilutions. (C) The mouse anti-GlgA antiserum labeling at 1:1000 dilution was further observed under a confocal microscope. Note that the intra-inclusion labeling with anti-GlgA polyclonal antibody displayed two distinct patterns [overlapping with chlamydial organisms (yellow arrowheads) and free of the organisms (red arrowheads)] while all anti-HSP60 antibody labelings overlapped with the organisms (panels d-f). The images were taken under an objective lens of either 100x (conventional fluorescence microscopy) or 60x (confocal microscopy).

**Figure 2:**
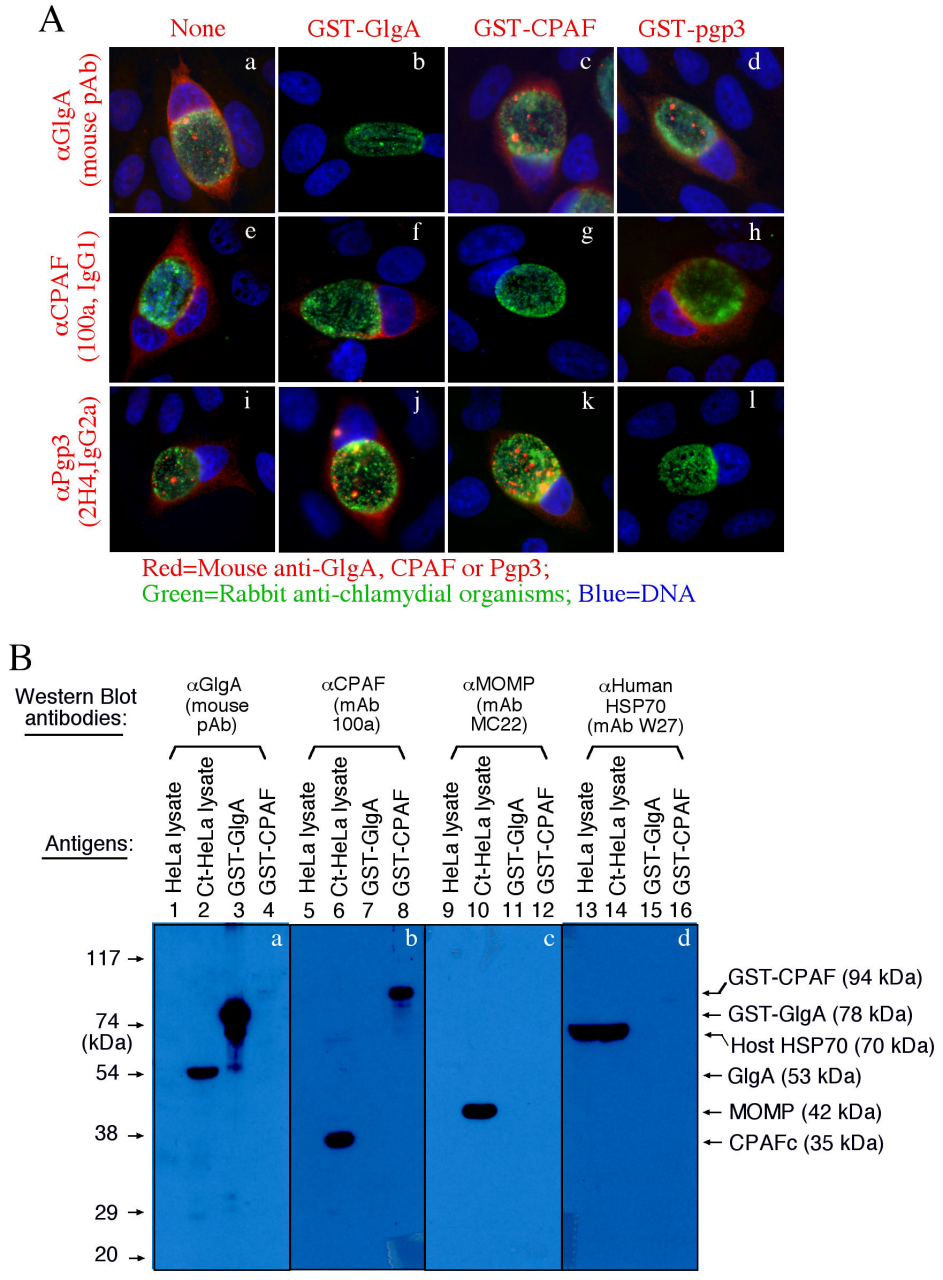
Antibody specificity validation. The mouse anti-GlgA (polycolonal antibody, pAb at 1:1000), anti-CPAF (monoclonal antibody, clone# 100a, IgG1) and anti-Pgp3 (clone# 2H4, IgG2a) antibodies were preabsorbed without (panels a, e & i) or with GST-GlgA (b, f & j), GST-CPAF (c, g & k) and GST-Pgp3 (d, h & l) fusion proteins prior to reacting with *C. trachomatis*-infected cultures as described in Figure 1 legend. Note that the antibody labelings were removed by the corresponding (f, g & k) but not irrelevant fusion proteins. (B) HeLa cell lysates, Chlamydia trachomatis (Ct)-infected HeLa cell lysates, GST-GlgA or GST-CPAF fusion proteins were resolved in SDS-polyacrylamide gel and transferred onto nitrocellulose membrane for Western blot detection with mouse anti-GlgA (αGlgA at 1:4000 dilution, panel a), αCPAF (mAb 100a, b), αMOMP (mAb MC22, c) and αhuman HSP70 (mAb W27, d). Note that these antibodies only detected their corresponding antigens without any significant cross-reactivity.

Mouse antisera made against 6 glycogen metabolism enzyme GST fusion proteins were used to label *C. trachomatis*-infected HeLa cells in an immunofluorescence assay. We found that only the endogenous GlgA was detected both inside and outside of chlamydial inclusions when the infected cultures were observed either 24h or 40h after infection (Figure 1A), suggesting that a portion of GlgA is secreted into host cell cytosol. When the anti-GST-GlgA antiserum was carefully titrated (B) and analyzed using confocal microscopy (C), we found that GlgA detected inside chlamydial inclusions displayed two distinct patterns with or without overlapping with chlamydial organisms, suggesting that a portion of GlgA is secreted out of chlamydial organisms into the lumen of chlamydial inclusions prior to accessing to host cell cytosol. The host cytosolic labeling pattern of GlgA was similar to those of CPAF, a known Chlamydia-secreted protease, and Pgp3, a known Chlamydia-secreted plasmid protein, respectively (Figure 2A).

Both the granular staining inside inclusion and the diffused staining in host cell cytosol by anti-GlgA antibody were removed by pre-absorption with GST-GlgA but not GST-CPAF or Pgp3 fusion proteins (Figure 2A). The same were true for anti-CPAF and Pgp3 stainings, demonstrating that the anti-GlgA, anti-CPAF and anti-Pgp3 antibodies specifically labeled the corresponding endogenous proteins without cross-reacting with each other. On a Western blot (Figure 2B), the mouse anti-GlgA antiserum only reacted with the endogenous GlgA and the GST-GlgA fusion protein without cross-reacting with any other proteins from *C. trachomatis*-infected cells or unrelated fusion proteins. The anti-CPAF mAb 100a detected both the GST-CPAF fusion protein and the C-terminal fragment of activated CPAF (CPAFc) in Chlamydia-infected cells as demonstrated previously [22]. As loading controls, the antibody specifically recognizing the chlamydial major outer membrane protein (MOMP) detected MOMP in the infected cell sample while the anti-human HSP70 antibody recognized HSP70 in both normal and infected HeLa samples. These results further confirmed that both anti-GlgA and anti-CPAF antibodies only specifically detected the corresponding endogenous proteins without cross-reacting with each other or any unrelated chlamydial or host cell proteins. Thus, we can conclude that the signals labeled by the anti-GlgA and anti-CPAF antibodies revealed under immunofluorescence microscope represent the corresponding endogenous proteins.

**Figure 3:**
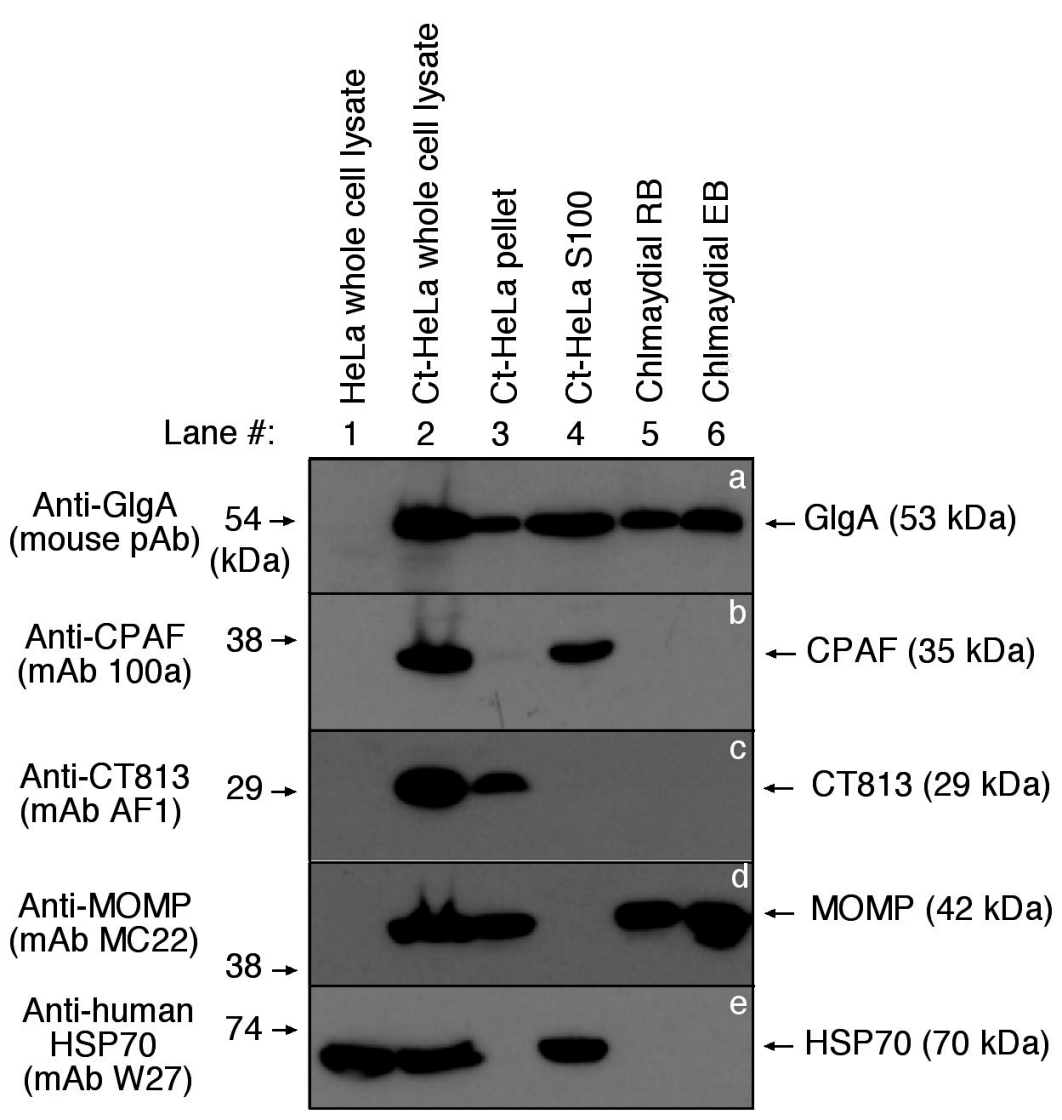
Western blot detection of GlgA in fractions of *C. trachomatis*-infected cells. Normal HeLa lysates (lane 1) or HeLa cells with Chlamydia trachomatis (Ct) serovar L2 infection (lane 2) were fractionated into pellets (lane 3) and supernatants (S100, lane 4) along with purified chlamydial reticulate bodies (RB, lane 5) or elementary body (EB, lane 6) were resolved in SDS polyacrylamide gel and the resolved protein bands were transferred onto nitrocellulose membrane for Western blot detection with anti-GlgA (panel a), anti-CPAF (b), anti-CT813 (c), anti-MOMP (d) and anti-human HSP70 (e) antibodies. Note that CPAF was detected only in either Ct-HeLa lysates (lane 2) or Ct-HeLa S100 (lane 4) while GlgA was detected in all samples containing chlamydial organisms or derived from *Chlamydia trachomatis*-infected cells.

The intracellular distribution of GlgA is also confirmed using a cell fractionation plus Western blot detection approach (Figure 3). CPAF was only detected in either the *C. trachomatis*-infected whole cell lysate (Ct-HeLa) or cytosolic fraction (Ct-HeLa S100) samples but not other samples including the purified *C. trachomatis* RB and EB organisms (pane b), which is consistent with what has been described previously [22]. However, GlgA was detected in all but normal HeLa cell samples (panel a), which is consistent with the observation that GlgA is associated with chlamydial organisms and secreted into both the inclusion lumen and host cell cytosol. To monitor the quality of the fractionation, the anti-CT813 (a known inclusion membrane protein; ref [29]:) and anti-MOMP antibodies were used to indicate the pellet fraction containing the chlamydial inclusions while an anti-human HSP70 antibody was used to indicate the host cell cytosolic fraction containing the *C. trachomatis*-secreted proteins. Detection with these antibodies revealed no cross contamination between the pellet and cytosolic fractions. In addition, detection with the anti-MOMP antibody showed that the amounts of chlamydial organisms in the *C. trachomatis*-infected HeLa whole cell lysate, the pellet fraction and purified EB and RB samples were equivalent. These results together have independently confirmed that chlamydial GlgA has a wide distribution in the infected cells.

### 2: Characterization of GlgA secretion

**Figure 4:**
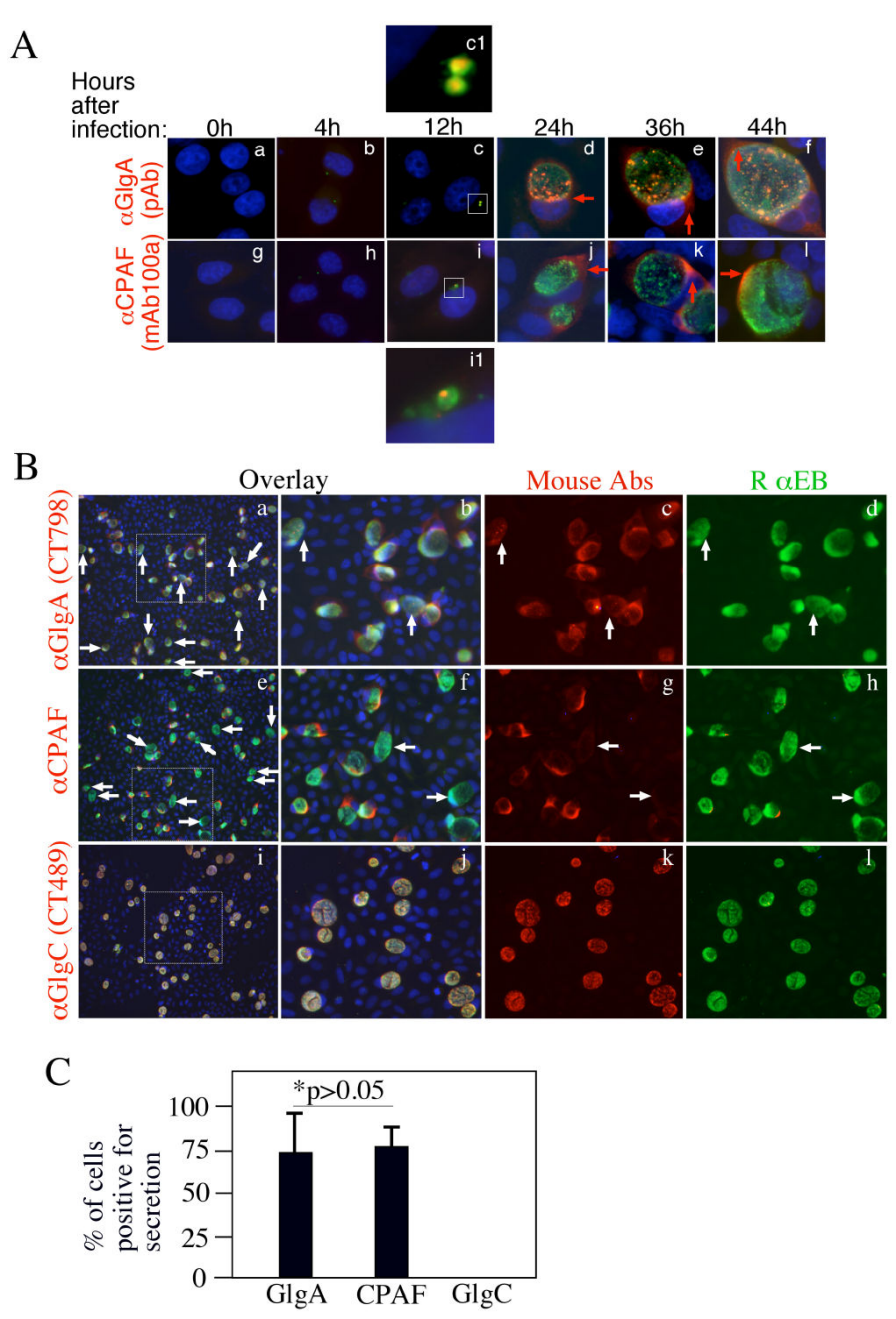
Time course expression and secretion of GlgA during *C. trachomatis* infection. HeLa cell infected with *C. trachomatis* serovar L2 at MOI of 0.5 were processed at various times after infection (as indicated on the top) for immunofluorescence staining as described in the legend to Figure 1. The labelings of anti-GlgA (polyclonal antiserum, panels a to f) and anti-CPAF (mAb 100a; g to l) were visualized with a goat anti-mouse IgG conjugated with Cy3 (red) while the rabbit anti-chlamydial organism labelings were visualized with a goat anti-rabbit IgG-Cy2 conjugate (green). Images amplified in separate panels were marked with white squares and the corresponding amplified images were labeled the same letters followed by the number 1. Note that both GlgA (c & c_1_) and CPAF (i & i1) proteins were first detected associated with the chlamydial inclusions at 12h while secretion out of the inclusions was first detected at 24h (panel d for GlgA; j for CPAF) post infection (red arrows). The secreted GlgA and CPAF remained in the infected cells throughout the infection cycle. The % of cells positive for GlgA (CT798) secretion into host cell cytoplasm was compared to those positive for CPAF secretion (B & C). HeLa cells infected with L2 for 40h were processed and triple labeled with mouse antibodies (red) against GlgA (CT798), CPAF or GlgC (CT489) in combination with a rabbit antibody against chlamydial organisms (green) and a DNA dye (blue). The images were taken under a 20X objective lens. The areas marked with dashed squares in images a, e & i were magnified in the corresponding panels on the right. White arrows point to inclusions without secretion of GlgA (images a-d) or CPAF (e–h) into host cell cytosol. GlgC was used as a secretion-negative control (panels i to l). The number of cells with positive secretion was counted and calculated into % of secretion-positive cells (C). The results were expressed as mean plus standard deviation and data came from 4 independent experiments. Please note that the % of cells secreting either GlgA or CPAF were similar (p>0.05, Fisher Exact).

**Figure 5:**
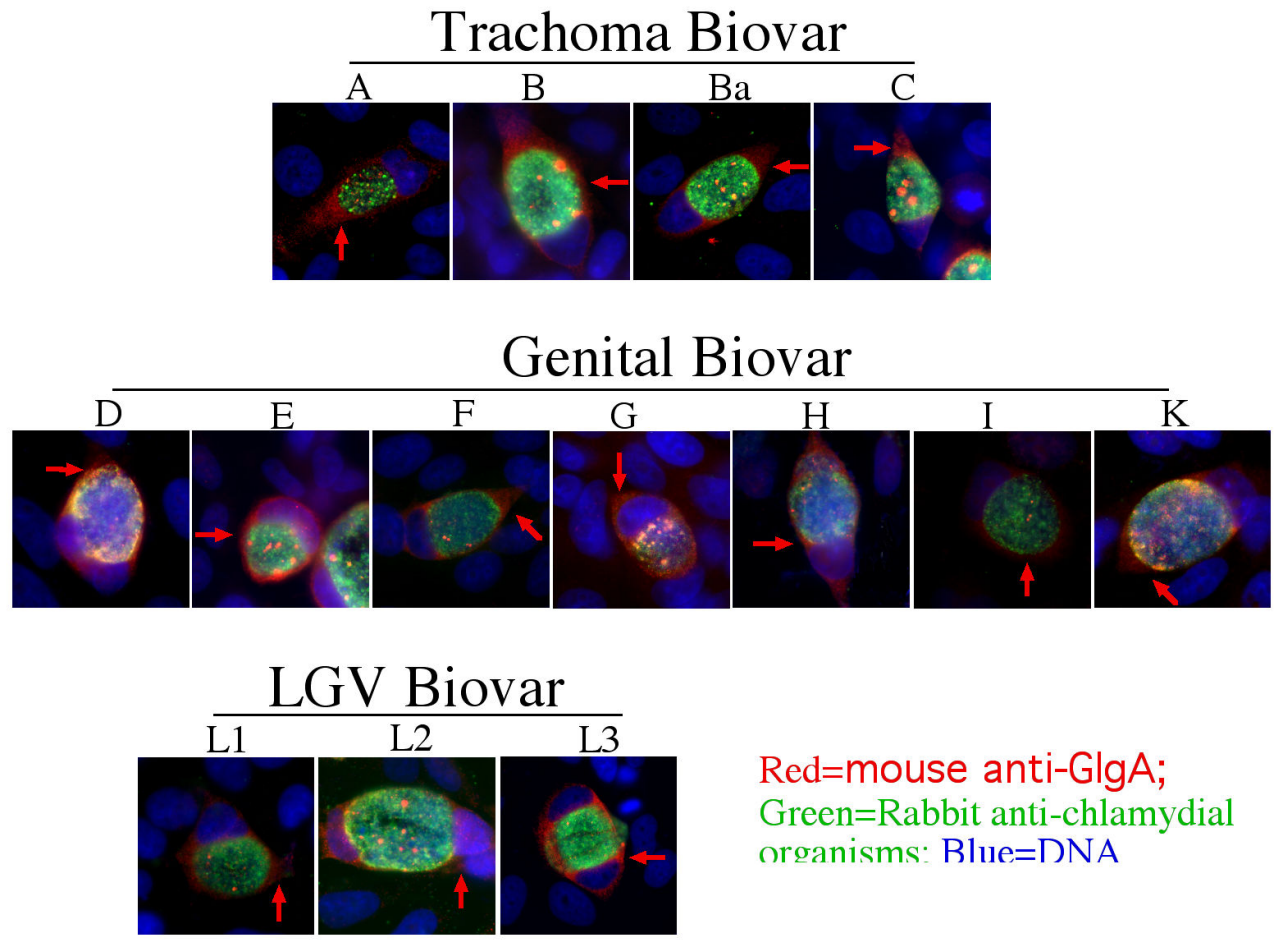
Secretion of GlgA into host cytosol by different biovars/serovars of *Chlamydia trachomatis*. HeLa cells were infected with different serovars of chlamydial organisms representing 3 major biovars of *Chlamydia trachomatis* as indicated on top of each panel. The infected cultures were subjected to immunofluorescence labelings as described in Figure 4 legend. Note that GlgA secretion into host cell cytosol was detected in all *Chlamydia trachomatis* serovars assayed.

**Figure 6:**
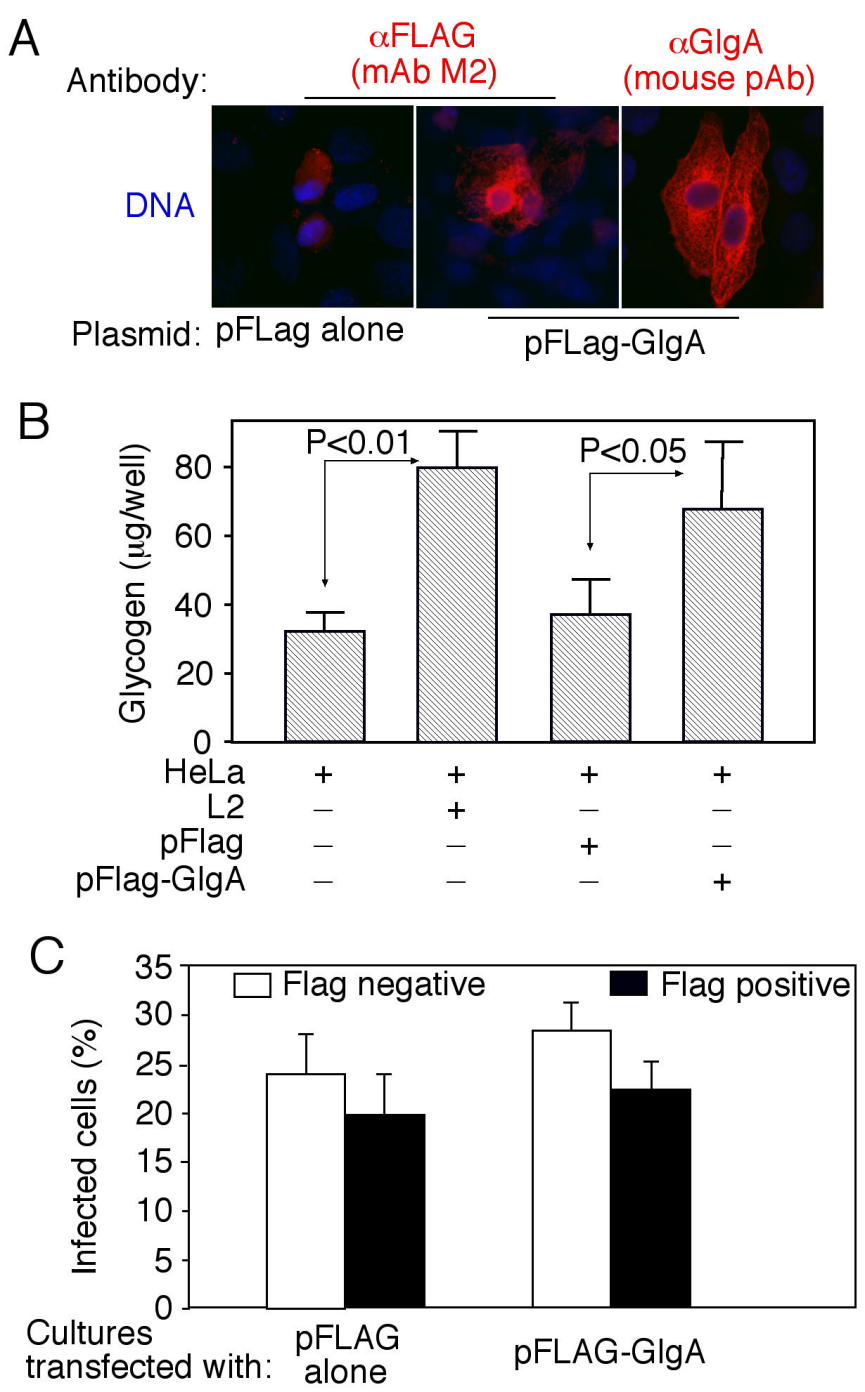
Effect of chlamydial GlgA expression on glycogen synthesis and subsequent chlamydial infection. HeLa cells were transfected with pFlag plasmid alone or pFlag plasmid encoding chlamydial GlgA gene. Twenty-four hours after transfection, the cell samples were processed for immunofluorescence labelings with mouse anti-FLAG or anti-GlgA (A), harvested for glycogen detection (B) or further infected with *C. trachomatis* L2 (C). (A) The images were taken 24h after transfection under a 60X objective lens using a conventional fluorescence microscope. (B) HeLa cells with or without transfection with pFLAG or pFLAG-GlgA or infection with L2 were harvested 24h post-transfection or 48h post-infection for glycogen measurements. The results coming from 5 independent experiments were expressed as µg per ml of samples as shown along the Y-axis. Note that both exogenous GlgA overexpression and chlamydial infection significantly enhanced the levels of glycogen synthesis in HeLa cells (Student t-test). (C) Some of the transfected cells were further infected with chlamydial organisms and 40h after chlamydial infection, the cultures were processed for immunofluorescence labelings with mouse anti-FLAG to identify positive transfection cells and rabbit anti-chlamydial EBs to identify chlamydial inclusions. Number of total and *C. trachomatis*-infected cells was counted from 5 random views of each coverslip. The infection rate was calculated for anti-Flag positive (solid bar) and negative (open bar) cells respectively and expressed as mean plus/minus standard deviation along the Y-axis. Note that the infection rates displayed no statistically significant differences between cells with or without transfection from either pFLAG vector alone or pFLAG/GlgA recombinant plasmid-transfected cultures (p>0.05, Fisher Exact).

We further used the specific anti-GlgA antibody to monitor biosynthesis and secretion of GlgA at single cell level (Figure 4). As shown in panel A, GlgA was first detected 12h post infection. Clear secretion into host cell cytosol was detected 24h after infection. These expression and distribution patterns were similar to those of CPAF with the exception that some GlgA molecules were obviously accumulated in organism-free granules in the lumen of chlamydial inclusions throughout the infection course. CPAF secretion into host cell cytosol was more complete without any significant accumulation in the lumen of inclusions. We further found that about 75% of the infected cells secreted GlgA or CPAF (Figure 4B & C). Secretion of GlgA into the chlamydial inclusion lumen and host cell cytosol was detected in all *C. trachomatis* biovar-infected cells (Figure 5), suggesting that GlgA secretion may represent an essential process required by all *C. trachomatis* organisms. To test whether GlgA secretion into host cell cytosol can impact chlamydial intracellular infection, we expressed GlgA via a transgene in HeLa cells and examined the effect of the preexisting GlgA in the host cell cytosol on subsequent chlamydial infection (Figure 6). Expression of chlamydial GlgA in HeLa cells (A) resulted in elevated levels of glycogen while the vector plasmid alone transfection failed to do so (B), suggesting that chlamydial GlgA expressed in the host cell cytosol is functional. However, HeLa cells with or without GlgA expression were similarly susceptible to the subsequent chlamydial infection (C), suggesting that neither the expressed GlgA nor the elevated level of glycogen in HeLa cells has any impact on chlamydial infection.

### 3: Chlamydial GlgA is immunogenic during chlamydial infection in women

**Figure 7:**
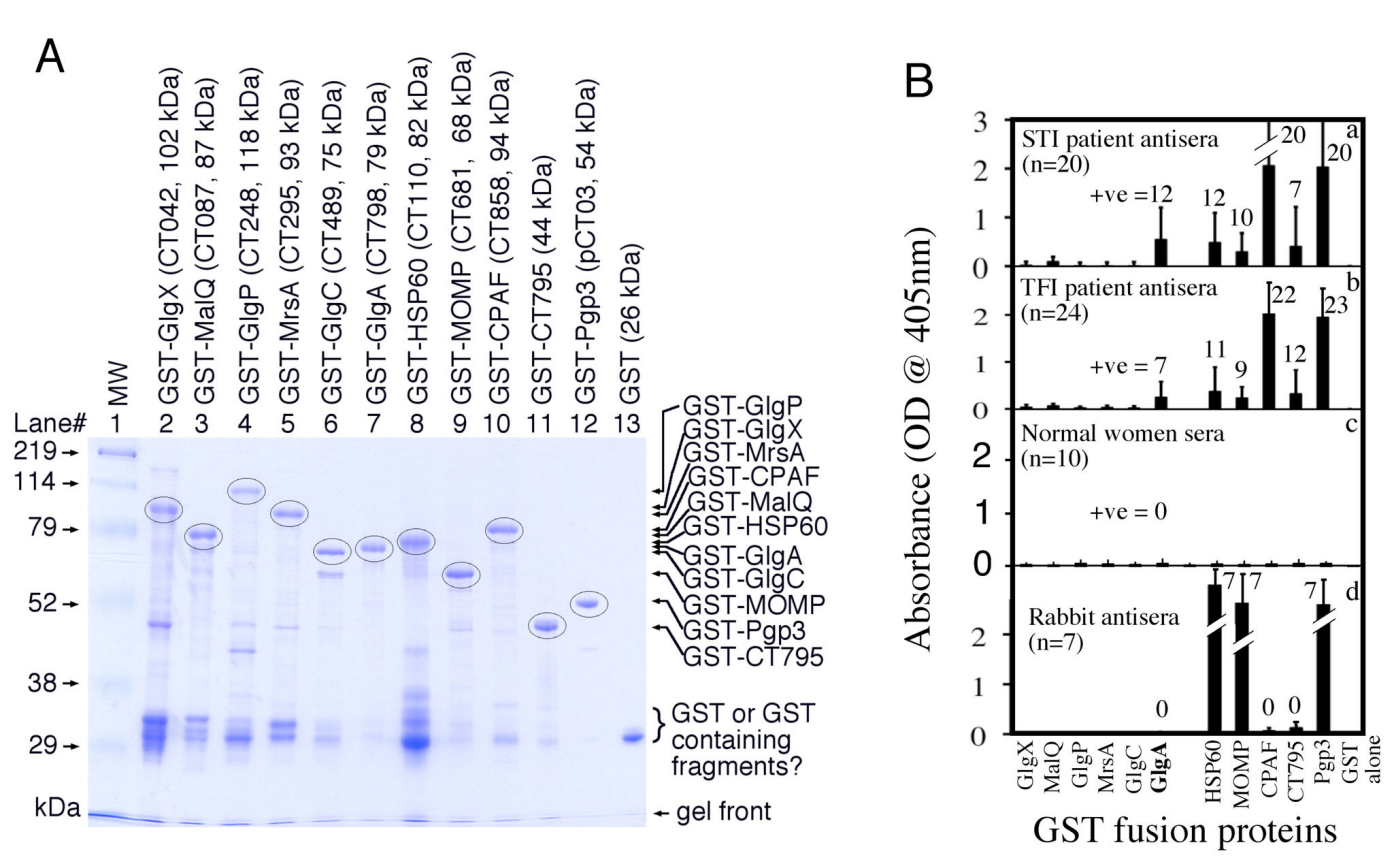
Reactivity of GlgA with antisera from women infected and rabbits immunized with *Chlamydia trachomatis* organisms. (**A**) Chlamydial GST fusion proteins or GST alone were resolved in a SDS polyacrylamide gel and stained with a Coomassie blue dye. All GST fusion proteins along with their gene names/ORF numbers and fusion protein molecular weights were listed on top of the gel image. Note that besides the GST-containing degradation fragments, the major bands are full length fusion proteins (marked with circles) for all GST fusion proteins. (B) The above fusion proteins listed along the X-axis were reacted with antisera from women diagnosed with acute *Chlamydia trachomatis* infection (STI patients, n=20, panel a) or tubal factor infertility (TFI patients, n=24, b) or from normal women without *C. trachomatis* infection (n=10, c) or from rabbits intramuscularly immunized with dead *Chlamydia trachomatis* organisms (Rabbits, n=7, c) in ELISA assays. The OD values obtained at the wavelength of 405nm in the format of mean plus/minus standard deviation were displayed along the Y-axis. Any reactivity with an OD value equal to or above the mean plus 2 standard deviations is defined as positive (+ve), which is noted on top of each bar. Note that 12 of the 20 STI, 7 of the 24 TFI and 0 of the 10 normal women or 7 rabbit antisera positively reacted with GlgA.

Since it is known that *C. trachomatis*-secreted proteins such as CPAF [30,35], Pgp3 [18] and CT795 [20] are highly immunogenic during chlamydial infection, we compared the immunogenicity of GlgA and other glycogen metabolism-related chlamydial proteins along with various control chlamydial proteins in both women urogenitally infected with *C. trachomatis* and rabbits intramuscularly immunized with dead *C. trachomatis* organisms (Figure 7). These fusion proteins were monitored for quality on a Coomassie blue-staining gel (A) prior to reacting with serum samples (B). GlgA was recognized by antisera from women diagnosed with either acute *C. trachomatis* infection (STI patients) or tubal factor infertility (TFI patients) with a recognition frequency of 60% or 29% respectively, suggesting that anti-GlgA antibodies are associated with acute *C. trachomatis* infection. However, the secreted proteins CPAF, Pgp3 and CT795 did not display such a preference with a recognition frequency of 100%, 95% & 35% by STI antisera and 92%, 96% & 50% by TFI antisera respectively. None of the 10 normal women reacted significantly with any of the chlamydial fusion proteins. When the chlamydial fusion proteins were reacted with 7 rabbits antisera, 100% recognition frequency was detected for chlamydial HSP60, MOMP and Pgp3 while no reactivity was detected for GlgA, CPAF or CT795. Since Pgp3 is also localized in chlamydial outer membrane, it is not surprising that Pgp3, like MOMP, is highly immunogenic when the dead EBs were used to immunize rabbits. However, GlgA is also associated with the chlamydial organisms but it failed to induce any antibody responses in rabbits. The observation that none of the other glycogen metabolism-related proteins were immunogenic following either chlamydial infection in humans or immunization in rabbits suggests that the organism-associated glycogen metabolism proteins are not immunogenic.

## Discussion

*Chlamydia trachomatis* has evolved strategies for secreting proteins into host cell cytosol, which may benefit chlamydial intracellular living. Identifying *C. trachomatis*-secreted proteins (CtSPs) represent an essential first step for uncovering the mysteries of the chlamydial intracellular life and understanding the molecular mechanisms of *C. trachomatis* pathogenesis. Here, we have presented evidence that the chlamydial glycogen synthase GlgA is secreted into host cell cytosol during *C. trachomatis* infection. First, the anti-GlgA antiserum detected GlgA outside of the chlamydial inclusions in *C. trachomatis*-infected cells. The extra-inclusion GlgA molecules displayed a cytosolic distribution pattern similar to that of CPAF, a known Chlamydia-secreted protease. Second, the detection of the endogenous GlgA was specific as demonstrated both in fluorescence microscopy and Western blot assays. Third, GlgA secretion into inclusion lumen and host cell cytosol was also confirmed using a cell fractionation plus Western blot detection approach. Finally, like CPAF and CT795, GlgA also contains a N-terminal signal sequence. Although we have not characterized the functionality of the signal sequence, its presence suggests that GlgA, like many other CtSPs that contain N-terminal secretion sequence, may use a sec-dependent pathway to gain access to both inclusion lumen and host cell cytosol.

Although *C. trachomatis* GlgA and other glycogen-related enzymes are expected to carry out glycogen biosynthesis and metabolism in the chlamydial organisms, the role of GlgA secreted out of the organisms remains unknown. Glycogen is abundantly detected in the lumen of chlamydial inclusion under electron microscopy [27,28]. Our current finding that GlgA is also localized in the lumen suggests that either chlamydial glycogen along with its synthase is transported into the lumen from the organisms or a portion of glycogen is synthesized in the lumen. More careful studies are required for accurately identifying the sites of glycogen biosynthesis. The next question is the role of GlgA secreted into host cytosol. Transient expression of chlamydial GlgA in the host cells resulted in an elevated level of glycogen. However, neither the preexisting GlgA nor the elevated glycogen in the host cell cytosol affected the subsequent chlamydial infection (see Figure 6), suggesting that Chlamydia-enhanced biosynthesis of glycogen in host cell cytosol serves no apparent advantage for chlamydial intracellular life. Efforts are underway to further elucidate the biological significance of GlgA secretion into host cell cytosol.

Among all the glycogen-related enzymes analyzed, only GlgA is immunogenic during chlamydial infection in humans, correlating the unique localization of GlgA in host cell cytosol since secretion of chlamydial proteins into host cell cytosol is known to enhance the immunogenicity of the secreted proteins [14,18,20,30,31,35,36]. The lack of immunogenicity of all analyzed chlamydial glycogen-related enzymes in rabbits when inactivated chlamydial organisms were used as immunogens suggests that the chlamydial organism-associated glycogen enzymes are immune recessive, which may be due to their inaccessibility to immune system when configured in the organisms. However, we previously showed that the glycogen metabolism enzymes were immunogenic when injected into mice [37]. Immunization with *C. muridarum* glycogen phosphorylase (GlgP) but not with the other glycogen metabolism enzymes induced a significant protection against intravaginal infection with *C. muridarum* organisms, suggesting that GlgP is immunogenic both during immunization and *C. muridarum* infection. However, it is not known whether *C. trachomatis* GlgP is immunogenic in mice. Nevertheless, the immunogenicity of *C. trachomatis* GlgA in women urogenitally infected with *C. trachomatis* indicates that GlgA is expressed and possibly secreted into host cell cytosol during chlamydial infection. Plasmid-free chlamydial organisms with a significantly reduced level of GlgA and lack of glycogen synthesis are highly attenuated in animal models [26,28], suggesting that the secreted *C. trachomatis* GlgA may contribute to *C. trachomatis* pathogenesis in humans.

## References

[1] Sherman KJ, Daling JR, Stergachis A, Weiss NS, Foy HM et al. (1990) Sexually transmitted diseases and tubal pregnancy. Sex Transm Dis 17: 115-121. doi:10.1097/00007435-199007000-00001. PubMed: 2247800. 2247800 10.1097/00007435-199007000-00001

[2] Peterman TA, Tian LH, Metcalf CA, Satterwhite CL, Malotte CK et al. (2006) High incidence of new sexually transmitted infections in the year following a sexually transmitted infection: a case for rescreening. Ann Intern Med 145: 564-572. doi:10.7326/0003-4819-145-8-200610170-00005. PubMed: 17043338. 17043338 10.7326/0003-4819-145-8-200610170-00005

[3] Centers for Disease Control and Prevention C (November 2009) Sexually Transmitted Disease Surveillance (2008). In: Services USDoHaH, editor. Atlanta, GA: http://www.cdc.gov/std/stats08/toc.htm.

[4] Stephens RS (2003) The cellular paradigm of chlamydial pathogenesis. Trends Microbiol 11: 44-51. doi:10.1016/S0966-842X(02)00011-2. PubMed: 12526854. 12526854 10.1016/s0966-842x(02)00011-2

[5] Zhong G (2009) Killing me softly: chlamydial use of proteolysis for evading host defenses. Trends Microbiol 17: 467-474. doi:10.1016/j.tim.2009.07.007. PubMed: 19765998. 19765998 10.1016/j.tim.2009.07.007 PMC2755597

[6] Cheng W, Shivshankar P, Li Z, Chen L, Yeh IT et al. (2008) Caspase-1 contributes to Chlamydia trachomatis-induced upper urogenital tract inflammatory pathologies without affecting the course of infection. Infect Immun 76: 515-522. doi:10.1128/IAI.01064-07. PubMed: 18025098. 18025098 10.1128/IAI.01064-07 PMC2223466

[7] Chen L, Lei L, Chang X, Li Z, Lu C et al. (2010) Mice deficient in MyD88 Develop a Th2-dominant response and severe pathology in the upper genital tract following Chlamydia muridarum infection. J Immunol 184: 2602-2610. doi:10.4049/jimmunol.0901593. PubMed: 20124098. 20124098 10.4049/jimmunol.0901593

[8] Hackstadt T, Fischer ER, Scidmore MA, Rockey DD, Heinzen RA (1997) Origins and functions of the chlamydial inclusion. Trends Microbiol 5: 288-293. doi:10.1016/S0966-842X(97)01061-5. PubMed: 9234512. 9234512 10.1016/S0966-842X(97)01061-5

[9] Clifton DR, Fields KA, Grieshaber SS, Dooley CA, Fischer ER et al. (2004) A chlamydial type III translocated protein is tyrosine-phosphorylated at the site of entry and associated with recruitment of actin. Proc Natl Acad Sci U S A 101: 10166-10171. doi:10.1073/pnas.0402829101. PubMed: 15199184. 15199184 10.1073/pnas.0402829101 PMC454183

[10] Engel J (2004) Tarp and Arp: How Chlamydia induces its own entry. Proc Natl Acad Sci U S A 101: 9947-9948. doi:10.1073/pnas.0403633101. PubMed: 15226494. 15226494 10.1073/pnas.0403633101 PMC454194

[11] Hower S, Wolf K, Fields KA (2009) Evidence that CT694 is a novel Chlamydia trachomatis T3S substrate capable of functioning during invasion or early cycle development. Mol Microbiol 72: 1423-1437. doi:10.1111/j.1365-2958.2009.06732.x. PubMed: 19460098. 19460098 10.1111/j.1365-2958.2009.06732.x PMC2997736

[12] Scidmore MA, Fischer ER, Hackstadt T (2003) Restricted fusion of Chlamydia trachomatis vesicles with endocytic compartments during the initial stages of infection. Infect Immun 71: 973-984. doi:10.1128/IAI.71.2.973-984.2003. PubMed: 12540580. 12540580 10.1128/IAI.71.2.973-984.2003 PMC145390

[13] Rockey DD, Scidmore MA, Bannantine JP, Brown WJ (2002) Proteins in the chlamydial inclusion membrane. Microbes Infect 4: 333-340. doi:10.1016/S1286-4579(02)01546-0. PubMed: 11909744. 11909744 10.1016/s1286-4579(02)01546-0

[14] Li Z, Chen C, Chen D, Wu Y, Zhong Y et al. (2008) Characterization of fifty putative inclusion membrane proteins encoded in the Chlamydia trachomatis genome. Infect Immun 76: 2746-2757. doi:10.1128/IAI.00010-08. PubMed: 18391011. 18391011 10.1128/IAI.00010-08 PMC2423075

[15] Valdivia RH (2008) Chlamydia effector proteins and new insights into chlamydial cellular microbiology. Curr Opin Microbiol 11: 53-59. doi:10.1016/j.mib.2008.01.003. PubMed: 18299248. 18299248 10.1016/j.mib.2008.01.003

[16] Fields KA, Mead DJ, Dooley CA, Hackstadt T (2003) Chlamydia trachomatis type III secretion: evidence for a functional apparatus during early-cycle development. Mol Microbiol 48: 671-683. doi:10.1046/j.1365-2958.2003.03462.x. PubMed: 12694613. 12694613 10.1046/j.1365-2958.2003.03462.x

[17] Wu X, Lei L, Gong S, Chen D, Flores R et al. (2011) The chlamydial periplasmic stress response serine protease cHtrA is secreted into host cell cytosol. BMC Microbiol 11: 87. doi:10.1186/1471-2180-11-87. PubMed: 21527029. 21527029 10.1186/1471-2180-11-87 PMC3107777

[18] Li Z, Chen D, Zhong Y, Wang S, Zhong G (2008) The chlamydial plasmid-encoded protein pgp3 is secreted into the cytosol of Chlamydia-infected cells. Infect Immun 76: 3415-3428. doi:10.1128/IAI.01377-07. PubMed: 18474640. 18474640 10.1128/IAI.01377-07 PMC2493211

[19] Gong S (2011) Chlamydia trachomatis secretion of hypothetical protein CT622 into host cell cytoplasm via a secretion pathway that can be inhibited by a C1 compound. Microbiology. 10.1099/mic.0.047746-0 PMC3139441 21233161

[20] Qi M, Lei L, Gong S, Liu Q, Delisa MP et al. (2011) Chlamydia trachomatis Secretion of an Immunodominant Hypothetical Protein (CT795) into Host Cell Cytoplasm. J Bacteriol 193: 2498-2509. doi:10.1128/JB.01301-10. PubMed: 21441519. 21441519 10.1128/JB.01301-10 PMC3133157

[21] Lei L, Qi M, Budrys N, Schenken R, Zhong G (2011) Localization of Chlamydia trachomatis hypothetical protein CT311 in host cell cytoplasm. Microb Pathog 51: 101-109. doi:10.1016/j.micpath.2011.05.002. PubMed: 21605656. 21605656 10.1016/j.micpath.2011.05.002 PMC3120901

[22] Zhong G, Fan P, Ji H, Dong F, Huang Y (2001) Identification of a chlamydial protease-like activity factor responsible for the degradation of host transcription factors. J Exp Med 193: 935-942. doi:10.1084/jem.193.8.935. PubMed: 11304554. 11304554 10.1084/jem.193.8.935 PMC2193410

[23] Gilkes MJ, Smith CH, Sowa J (1958) Staining of the inclusion bodies of trachoma and inclusion conjunctivitis. Br J Ophthalmol 42: 473-477. doi:10.1136/bjo.42.8.473. PubMed: 13572759. 13572759 10.1136/bjo.42.8.473 PMC509682

[24] Chun Y, Yin ZD (1998) Glycogen assay for diagnosis of female genital Chlamydia trachomatis infection. J Clin Microbiol 36: 1081-1082. PubMed: 9542941. 9542941 10.1128/jcm.36.4.1081-1082.1998 PMC104693

[25] Stephens RS, Kalman S, Lammel C, Fan J, Marathe R et al. (1998) Genome sequence of an obligate intracellular pathogen of humans: Chlamydia trachomatis. Science 282: 754-759. doi:10.1126/science.282.5389.754. PubMed: 9784136. 9784136 10.1126/science.282.5389.754

[26] O’Connell CM, Ingalls RR, Andrews CW Jr., Scurlock AM, Darville T (2007) Plasmid-deficient Chlamydia muridarum fail to induce immune pathology and protect against oviduct disease. J Immunol 179: 4027-4034. PubMed: 17785841. 17785841 10.4049/jimmunol.179.6.4027

[27] Chiappino ML, Dawson C, Schachter J, Nichols BA (1995) Cytochemical localization of glycogen in Chlamydia trachomatis inclusions. J Bacteriol 177: 5358-5363. PubMed: 7545158. 7545158 10.1128/jb.177.18.5358-5363.1995 PMC177334

[28] Carlson JH, Whitmire WM, Crane DD, Wicke L, Virtaneva K et al. (2008) The Chlamydia trachomatis plasmid is a transcriptional regulator of chromosomal genes and a virulence factor. Infect Immun 76: 2273-2283. doi:10.1128/IAI.00102-08. PubMed: 18347045. 18347045 10.1128/IAI.00102-08 PMC2423098

[29] Chen C, Chen D, Sharma J, Cheng W, Zhong Y et al. (2006) The hypothetical protein CT813 is localized in the Chlamydia trachomatis inclusion membrane and is immunogenic in women urogenitally infected with C. trachomatis. Infect Immun 74: 4826-4840. doi:10.1128/IAI.00081-06. PubMed: 16861671. 16861671 10.1128/IAI.00081-06 PMC1539634

[30] Wang J, Zhang Y, Lu C, Lei L, Yu P et al. (2010) A Genome-Wide Profiling of the Humoral Immune Response to Chlamydia trachomatis Infection Reveals Vaccine Candidate Antigens Expressed in Humans. J Immunol 185: 1670-1680. doi:10.4049/jimmunol.1001240. PubMed: 20581152. 20581152 10.4049/jimmunol.1001240

[31] Sharma J, Zhong Y, Dong F, Piper JM, Wang G et al. (2006) Profiling of human antibody responses to Chlamydia trachomatis urogenital tract infection using microplates arrayed with 156 chlamydial fusion proteins. Infect Immun 74: 1490-1499. doi:10.1128/IAI.74.3.1490-1499.2006. PubMed: 16495519. 16495519 10.1128/IAI.74.3.1490-1499.2006 PMC1418620

[32] Budrys NM, Gong S, Rodgers AK, Wang J, Louden C et al. (2012) Chlamydia trachomatis antigens recognized in women with tubal factor infertility, normal fertility, and acute infection. Obstet Gynecol 119: 1009-1016. doi:10.1097/AOG.0b013e3182519326. PubMed: 22525912. 22525912 10.1097/AOG.0b013e3182519326 PMC4608258

[33] Zhong Y, Weininger M, Pirbhai M, Dong F, Zhong G (2006) Inhibition of staurosporine-induced activation of the proapoptotic multidomain Bcl-2 proteins Bax and Bak by three invasive chlamydial species. J Infect 53: 408-414. doi:10.1016/j.jinf.2005.12.028. PubMed: 16490255. 16490255 10.1016/j.jinf.2005.12.028

[34] Fan T, Lu H, Hu H, Shi L, McClarty GA et al. (1998) Inhibition of apoptosis in chlamydia-infected cells: blockade of mitochondrial cytochrome c release and caspase activation. J Exp Med 187: 487-496. doi:10.1084/jem.187.4.487. PubMed: 9463399. 9463399 10.1084/jem.187.4.487 PMC2212145

[35] Sharma J, Bosnic AM, Piper JM, Zhong G (2004) Human antibody responses to a Chlamydia-secreted protease factor. Infect Immun 72: 7164-7171. doi:10.1128/IAI.72.12.7164-7171.2004. PubMed: 15557641. 15557641 10.1128/IAI.72.12.7164-7171.2004 PMC529132

[36] Qi M, Gong S, Lei L, Liu Q, Zhong G (2011) A Chlamydia trachomatis OmcB C-terminal fragment is released into host cell cytoplasm and is immunogenic in humans. Infect Immun. 10.1128/IAI.00003-11 PMC3125825 21422182

[37] Li Z, Lu C, Peng B, Zeng H, Zhou Z et al. (2012) Induction of protective immunity against Chlamydia muridarum intravaginal infection with a chlamydial glycogen phosphorylase. PLOS ONE 7: e32997. doi:10.1371/journal.pone.0032997. PubMed: 22427926. 22427926 10.1371/journal.pone.0032997 PMC3299733

